# Blockchain-Based Method for Spatial Retrieval and Verification of Remote Sensing Images

**DOI:** 10.3390/s24072078

**Published:** 2024-03-24

**Authors:** Yujie Liu, Yuanfei Chang

**Affiliations:** 1 Aerospace Information Research Institute, Chinese Academy of Sciences, Beijing 100094, China; liuyujie21@mails.ucas.ac.cn; 2 University of Chinese Academy of Sciences, Beijing 100049, China

**Keywords:** blockchain technology, spatial data indexing, hyperledger fabric, IPFS, remote sensing image protection

## Abstract

Remote sensing image is a vital basis for land management decisions. The protection of remote sensing images has seen the application of blockchain’s notarization function by many scholars. Yet, research on efficient retrieval of such images on the blockchain remains sparse. Addressing this issue, this paper introduces a blockchain-based spatial index verification method using Hyperledger Fabric. It linearizes the spatial information of remote sensing images via Geohash and integrates it with LSM trees for effective retrieval and verification. The system also incorporates IPFS as an underlying storage unit for Hyperledger Fabric, ensuring the safe storage and transmission of images. The experiments indicate that this method significantly reduces the latency in data retrieval and verification without impacting the write performance of Hyperledger Fabric, enhancing throughput and providing a solid foundation for efficient blockchain-based verification of remote sensing images in land registry systems.

## 1. Introduction

### 1.1. Background

Remote sensing image captures the actual visual information of the Earth’s surface and accurately reflects the current state of land and buildings, serving as a crucial basis for land management decisions. Considering the data consistency issues commonly encountered during storage, transfer, and application, finding an effective method to protect remote sensing image is vital. This ensures accurate usage, avoiding significant losses in land management decisions due to errors like incorrect land ownership determination and misguided planning. In this context, blockchain technology, originally the underlying technology for cryptocurrencies like Bitcoin, emerges as a powerful solution [1,2]. As a distributed database system, blockchain stores data in chronologically arranged blocks linked cryptographically. The immutable nature of blockchain, coupled with its decentralized storage across nodes and encryption techniques, offers high security, making it a potential solution for protecting the integrity and authenticity of remote sensing image while safeguarding against unauthorized access and data tampering [3,4].

Applying blockchain technology to the protection of remote sensing images is challenging due to the significant storage pressure it creates, as each node stores a complete data set [5]. To alleviate this, most blockchain networks control the size of the data being added [6]. Addressing both storage and efficient retrieval issues is crucial for integrating blockchain with remote sensing image protection [7]. This study introduces a blockchain-based method for remote sensing image retrieval verification. By combining the IPFS file system with Hyperledger Fabric (HLF) blockchain, it leverages IPFS’s decentralized storage to distribute the storage burden and improve data access efficiency. Additionally, the method employs Geohash spatial filling curves to optimize the spatial data representation of remote sensing images. This, combined with LSM trees, creates an efficient spatial data indexing system, enabling effective spatial data queries on the blockchain.

In this paper, the authors contribute the following:

1. A collaborative on-chain and off-chain method for storing remote sensing images is proposed. The IPFS file system is utilized as an off-chain storage unit under Hyperledger Fabric for storing actual remote sensing image data, while on-chain, verification information and pointers related to these images are recorded. This ensures secure data storage and efficient transmission.
2. The Geohash spatial filling curve is used to transform spatial data of remote sensing images into one-dimensional codes that are easier to manage and query. Combined with the LSM tree data structure, an efficient spatial data index for remote sensing images on Hyperledger Fabric is designed, enhancing both efficient management and rapid querying of spatial data.
3. A complete remote sensing image retrieval and verification system is deployed and tested for its on-chain spatial index performance. The experiments include assessments of key metrics like latency and throughput, demonstrating the system’s ability to reduce access delay and improve overall throughput in Hyperledger Fabric when handling spatial data.

### 1.2. Related Works

Currently, the primary methods for protecting remote sensing image data are categorized into digital signature technology and digital watermarking [8,9]. Digital signature technology is mainly used to verify data integrity and confirm the identity of the data sender [10]. In contrast, digital watermarking is typically employed to embed invisible identification information into images, enabling copyright tracking and proof in cases of unauthorized use. The security performance of digital signatures, highly effective in detecting even minor changes in remote sensing image, largely depends on key management and the choice of encryption algorithms. With the advancement of computing capabilities and the development of quantum computing, these technologies need continuous updates and maintenance to ensure their long-term security [9].

Digital watermarking technology embeds watermark information into digital data, making it an inseparable part of the data [11]. This technique can embed encrypted copyright information and user data into images, extractable when needed to ensure image authenticity [12]. While digital watermarking effectively protects the digital copyright of remote sensing images, its impact on image application quality has been a concern [13]. Modern watermarking techniques aim to minimize this negative impact. For example, a DWT-based algorithm for remote sensing images watermarks only the highest root mean square value sub-bands to reduce impact on image classification performance [14]. Concepts like zero-watermarking [15], which protect copyright without modifying the original data, and algorithms focusing on security, imperceptibility [16], and robustness against attacks [17] have been proposed. However, as image processing and deep learning technologies evolve, the security challenges for digital watermarking, such as watermark removal and attacks, become increasingly severe.

With the development of blockchain technology, scholars have started applying it to protect remote sensing images. Blockchain’s high security, decentralization, tamper-proof, and traceability features offer a new method for the protection and data security management of remote sensing images. Researchers like Yongfei Lv [8] have stored encrypted hash values of remote sensing images on the blockchain to protect digital copyright. Kaimeng Ding [18] proposed an authentication method combining subject-sensitive hash with blockchain for remote sensing images, focusing on image content’s main features for robust security. Despite blockchain’s storage limitations, Dingjie Xu [9] suggested a method integrating blockchain technology with perceptual hashing, storing the perceptual hash value of images in IPFS and recording the storage address on the blockchain. This approach effectively reduces the storage pressure on the blockchain, leveraging IPFS’s efficient storage capabilities. Compared to traditional digital signatures and watermarking, blockchain technology provides more comprehensive protection for remote sensing images, including copyright protection, ensuring data authenticity and integrity, and facilitating efficient tracking and management of data usage history. However, efficiently retrieving remote sensing images on the blockchain remains a crucial challenge.

## 2. Methods

**Figure 1:**
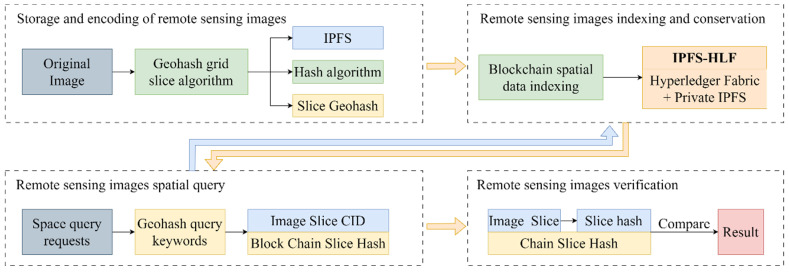
Blockchain-based method for retrieval and verification of remote sensing images.

This paper introduces a blockchain-based method for retrieving and verifying remote sensing images. The overall concept, illustrated in Figure 1, is composed of two main parts: a remote sensing image notarization method based on the IPFS-HLF (InterPlanetary File System-Hyperledger Fabric) architecture, and the construction of a spatial index for remote sensing images on the blockchain. The method combines a private IPFS network with Hyperledger Fabric to create a collaborative on-chain and off-chain storage system. The original data of remote sensing images are stored in the IPFS network, while content identifiers and SHA256 hash values of the images are stored in the Hyperledger Fabric network. The approach also designs a blockchain-based spatial index for remote sensing images to enhance the efficiency of on-chain verification in large-scale spatial data scenarios.

### 2.1. Remote Sensing Image Preservation Based on IPFS-HLF Architecture

**Figure 2:**
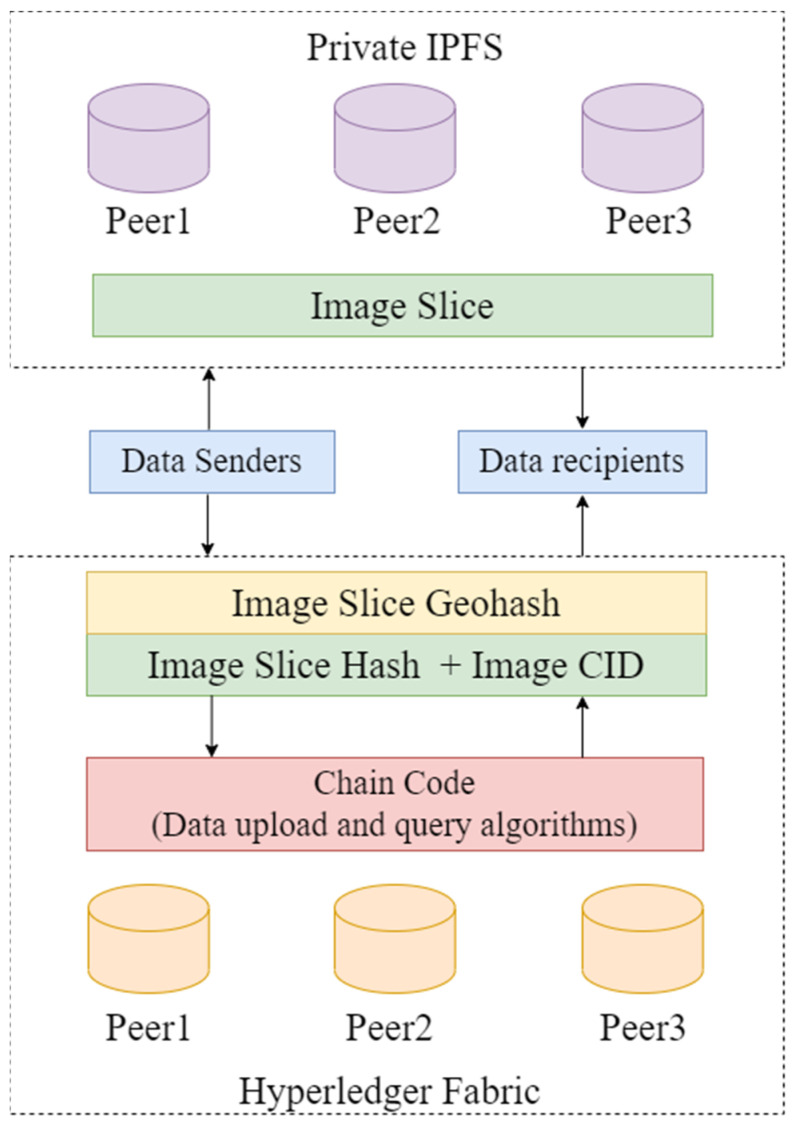
IPFS-HLF on-chain and off-chain collaborative storage architecture.

To effectively utilize blockchain for protecting remote sensing images, this section proposes a collaborative on-chain and off-chain notarization method based on the IPFS file system and Hyperledger Fabric consortium chain. This approach involves storing large volumes of original remote sensing image data in IPFS, while recording crucial metadata and hash summaries on the Hyperledger Fabric blockchain. This method successfully combines efficient storage with enhanced security, significantly improving the storage efficiency and safety of remote sensing images. The IPFS-HLF on-chain-off-chain cooperative storage architecture is shown in Figure 2.

#### 2.1.1. Off-Chain Storage of Remote Sensing Images Based on IPFS

Hyperledger Fabric, an open-source, permissioned enterprise blockchain platform [19], offers a highly customizable, secure, and modular architecture suitable for a variety of industry applications. It uses chaincode to manage the business logic of interactions within the blockchain network. In this research, to alleviate storage pressure on Hyperledger Fabric, remote sensing image data is not stored directly in it. Instead, a private IPFS network serves as an auxiliary off-chain storage unit, storing only the hash values of images and their addresses in the IPFS system. This ensures data immutability and increases storage efficiency. IPFS, a secure and efficient distributed storage and transfer protocol, uses content-addressing to build a hash-verified storage system. Combining IPFS with Hyperledger Fabric has proven more efficient, suitable for various data storage scenarios [20,21]. Remote sensing images stored in the private IPFS network are accessed uniquely via generated Content Identifiers (CIDs), which also enter the on-chain notarization process.

#### 2.1.2. On-Chain Preservation of Remote Sensing Images Based on Hyperledger Fabric

**Figure 3:**
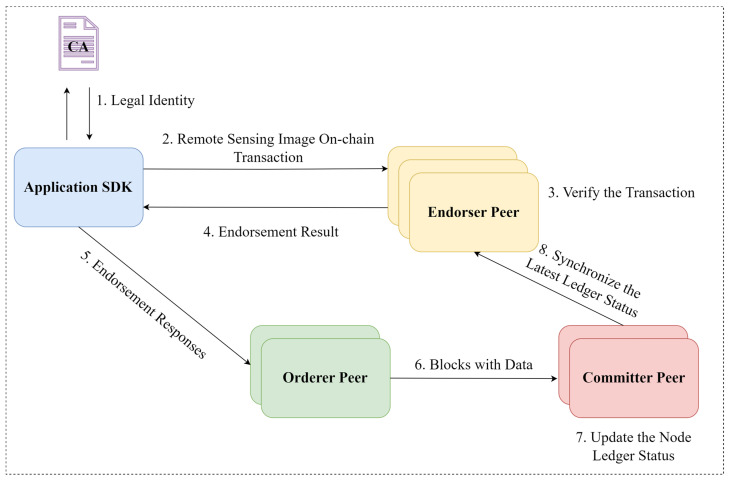
Remote sensing image depository process.

In the on-chain notarization phase for remote sensing image using Hyperledger Fabric, the process involves client nodes requesting certification from the consortium network via SDK to obtain legitimate access. Once authenticated, these nodes can connect to the network and utilize a specifically designed smart contract (chaincode) for the notarization of remote sensing images. This smart contract is responsible for securely storing the image content identifier (CID) and the integrity signature on the blockchain, ensuring the transparency, security, and immutability of the stored data. The entire procedure is illustrated in Figure 3.

After clients obtain legitimate identities from the Certification Authority (CA), they send encoded remote sensing images as transaction proposals to multiple endorsing nodes via SDK. The endorsers verify the proposal’s signatures and legality. Endorsing nodes simulate and return the execution result based on their ledger data and chaincode, which defines the transaction logic. The client collects and compares execution results from various endorsers to ensure the transaction is based on an unaltered ledger before submitting it to orderer nodes. Orderer sort transactions by time, creating blocks when certain conditions are met, which are then sent to committer nodes for integrity and legality checks. This mechanism ensures blockchain ledger consistency, updating all nodes with the finalized ledger, thus completing the on-chain process for remote sensing images.

### 2.2. Spatial Index Construction on Blockchain for Remote Sensing Images

Hyperledger Fabric’s structure for storing remote sensing image data includes two interconnected components: the blockchain ledger and the world state database (StateDB). The blockchain ledger logs every transaction that alters the state, while StateDB captures and accumulates the latest transaction states, keeping the data current. This design optimizes data retrieval by reducing disk I/O and avoiding the need for full ledger traversal during each query.

The LSM tree index model of LevelDB, utilized by most blockchain systems including the StateDB of Hyperledger Fabric [22], allows for rapid data writing in storage order, speeding up data retrieval during queries. However, LSM tree indexing is primarily suitable for one-dimensional key-value data, not multi-dimensional data like remote sensing images. Traditional blockchain methods store image hash summaries and use LSM tree indexing for keyword queries, which, while secure, are limited in spatial data querying and do not support remote sensing image retrieval based on spatial location or multi-dimensional features. Geohash converts two-dimensional coordinates into one-dimensional representations, dividing the Earth’s surface along longitude and latitude into non-overlapping grid cells with unique string encodings [23,24]. By combining Geohash with Hyperledger Fabric’s LSM tree in the world state database, this chapter constructs an on-chain spatial data index that enables efficient retrieval of geolocation-based remote sensing images on the Hyperledger Fabric platform, significantly enhancing spatial data processing efficiency.

#### 2.2.1. Coding of Remote Sensing Images

**Figure 4:**
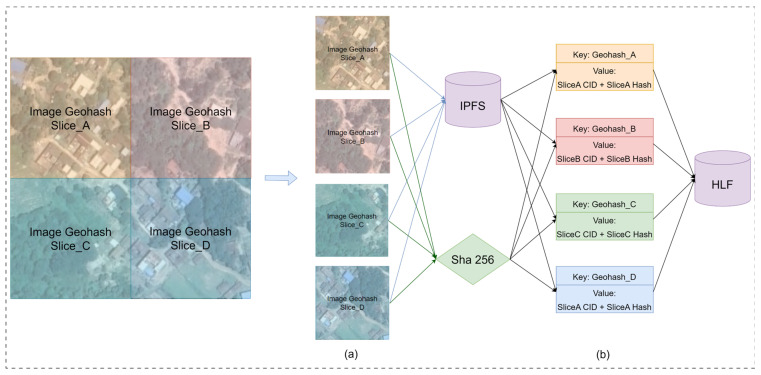
Coding process for remote sensing images. (**a**) Geohash grid-based image slicing; (**b**) Remote sensing image encoding.

**Figure 5:**
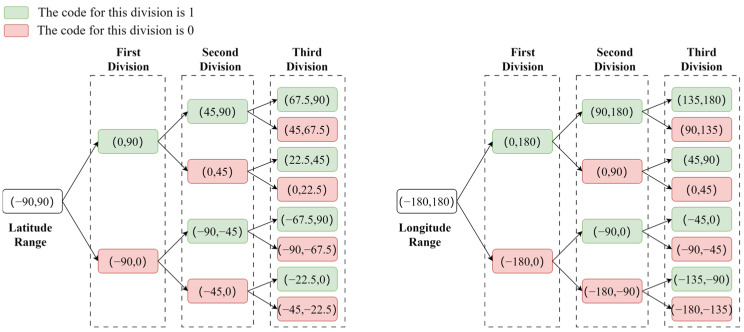
Grid interval division and binary encoding process.

Images need to be encoded and processed before they are uploaded to the blockchain. The remote sensing image encoding process is shown in Figure 4:

To precisely load the needed remote sensing images and reduce unnecessary disk transfers, a slicing method based on Geohash grids is employed. The generation of Geohash grids can be roughly divided into four processes: interval division, binary encoding of latitude and longitude, merging of latitude and longitude encodings, and conversion of binary encoding to Base32 encoding. The division of intervals requires determining the number of times the spatial grid is divided based on the length of the Geohash characters. For example, with a Geohash character length of 8, according to the subsequent Base32 encoding rules, the total number of divisions is five times the length of the encoding, corresponding to 20 divisions in the longitude direction and 20 divisions in the latitude direction. The longitude span of a Geohash grid with a length of 8 is 38.2m, and the corresponding latitude span is 19.1m. Following this step length, sampling of the spatial information of the image starts from the spatial coordinates of the lower left corner of the remote sensing image, and binary encoding is performed on the longitude and latitude of the sampling points based on their spatial location. Taking the coordinates of the lower left corner of the image (116.40382, 39.918118) as an example, the division and encoding process of the latitude and longitude intervals is shown in Figure 5. The longitude 116.40382 falls within the (0, 180) interval at the first division, so the encoding for the first division is 1. At the second division, it falls within the (90, 180) interval, so the second encoding is also 1. This process is repeated for a total of twenty divisions, resulting in a binary string of 116.40382 as 11010010110001101010. Similarly, the binary string for latitude 39.918118 after twenty divisions is 10111000110001011011.

**Figure 6:**
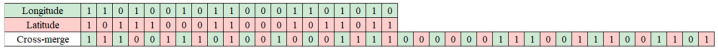
Binary string encoding merging process. The green in the figure represents the binary encoding of longitude, and the red represents the binary encoding of latitude.

The merging of the codes is accomplished by alternately merging the binary strings of longitude and latitude, resulting in a new binary string. Figure 6 demonstrates the process of merging these binary strings. The newly generated binary string fully integrates longitude and latitude information, ensuring that adjacent geographical locations also have similar prefixes in their encoding.

For ease of use and storage, the merged long binary string is converted into a shorter Base32 encoded string. This is done by dividing the long binary string into groups of five bits each, with each group corresponding to a Base32 character, resulting in the final Geohash encoding. The long binary string “11100 11101 00100 01111 00000 01110 01110 01101” is converted into the Geohash code “wx4g0ffe”. This code represents a Geohash grid that includes the lower left corner coordinates (116.40382, 39.918118) of the image, with the spatial information of the grid being represented by the Geohash string “wx4g0ffe”. The slicing method based on Geohash grids significantly divides the image into smaller units, using the corresponding Geohash code to represent the spatial location of each slice, thus enabling the system to more efficiently locate and load image data of specific areas. Once sliced, the image slices are stored in the off-chain IPFS system. The file content identifiers (CIDs) from IPFS, combined with the hash summaries of the image slices, form the pre-chain encoding.

The components of the code are shown in Figure 4b, where Geohash_A denotes the Geohash encoding of the spatial information of Slice_A, which is used as the key value of the on-chain index of Slice_A; CID is the storage address of the image in the IPFS file system; and Slice_A Hash is the hash digest of Slice_A encrypted by the SHA256 algorithm, which is used for verifying the authenticity of the slice.

#### 2.2.2. Construction of Spatial Indexes on the Blockchain

**Figure 7:**
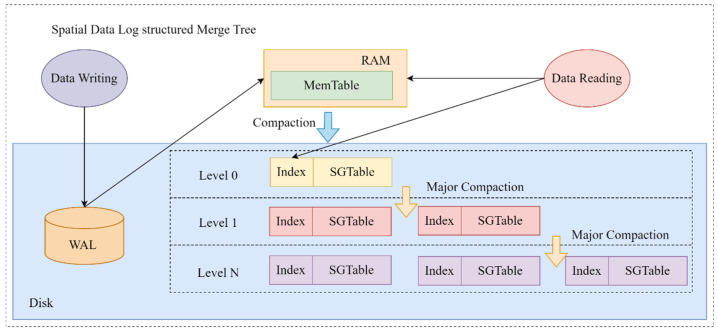
Hyperledger Fabric Spatial Index Tree based on LSM tree.

As illustrated in Figure 7, before writing encoded remote sensing image data into the state database, it is first logged in the Write-Ahead Log (WAL) to ensure data integrity. Then, the data is initially written to the Memtable layer, which offers faster access. When Memtable reaches a certain threshold, the Geohash-keyed image encoding data is organized into Sorted Geohash Tables (SGTables) and refreshed onto the disk. The disk space is segmented into layers with limited SGTables capacity, triggering a compaction process upon reaching capacity limits. This process optimizes storage usage, reduces redundant and outdated data, and, by reordering and organizing data, enhances data retrieval efficiency. SGTables data, sorted by Geohash characters, have a worst-case time complexity of O(n) for data retrieval. However, typically, efficient search algorithms like binary search are used, leveraging Geohash’s orderliness to significantly reduce search times and improve efficiency.

#### 2.2.3. On-Chain Remote Sensing Image Query and Verification

Leveraging the spatial proximity of remote sensing images on the disk, as processed in previous sections, this section introduces an on-chain spatial query algorithm based on the previously designed on-chain spatial index. The algorithm converts the spatial query area into a Geohash grid coverage area, using these grids’ Geohash codes as keywords to retrieve corresponding images from that region on the blockchain. This method utilizes the spatial organization of the data to enhance the efficiency and accuracy of querying remote sensing images.

**Figure 8:**
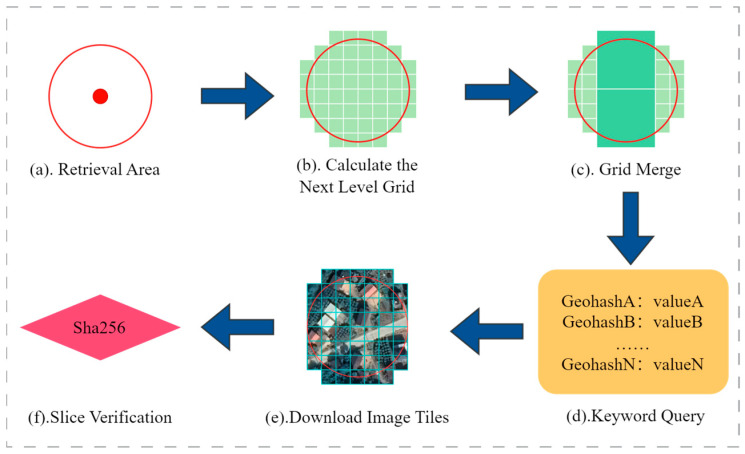
Steps for remote sensing image query and verification.

This method initially calculates all Geohash grids within the entire search range, based on the divided levels of remote sensing image slices. As shown in Figure 8c, an optimization strategy is employed to reduce query requests and enhance efficiency. The algorithm checks if the query area includes complete parent grids. If so, it uses the parent grids’ Geohash codes for queries instead of numerous child grid codes. This optimization reduces the number of requests while maintaining result completeness. After retrieving results, the algorithm downloads images from the storage address and compares their SHA256 hash summary with the on-chain hash to verify the images’ authenticity and integrity.

## 3. Experiment and Results

To evaluate the performance of the method proposed in this study, we constructed an application for batch execution of writing and querying remote sensing images. This evaluation followed the blockchain performance assessment method proposed by Pongnumkul [25], assessing the on-chain spatial index performance based on execution time, latency, and throughput. The values for execution time, latency, and throughput in the experiments represent the average outcomes from multiple runs. Additionally, the retrieval accuracy of the algorithm was evaluated using precision and recall rates [26,27]. The application’s functionalities include the following: batch writing of encoded remote sensing image data, querying a specified number of remote sensing images based on spatial range, and batch querying of remote sensing images by keywords.

### 3.1. Experimental Environment

The experiment was conducted using Hyperledger Fabric version v1.0. The Hyperledger Fabric network comprised two organizations, each contributing one peer node, with the ordering service running on an independent node of a third organization. A single channel was established between the two organizations, deploying both the Hyperledger Fabric official chaincode (Sample chaincode) and the spatial data index chaincode designed for this study. The ordering service operated with default parameters, a batch size of 500, and a batch timeout of 1 s. The nodes were deployed on two machines connected via a 1 Gbps switch, each equipped with an Intel Core i5-12600KF processor and 16 GB of memory.

### 3.2. Performance Data Collection

The experiment focused on evaluating the Hyperledger Fabric network’s data writing and querying performance without IPFS interaction, utilizing an on-chain spatial data indexing chaincode. Performance was assessed based on three metrics: execution time, latency, and throughput. This approach aimed to understand the efficiency of the Hyperledger Fabric network in handling spatial data indexing and retrieval tasks. To calculate these metrics, the dataset collected for each transaction included:

- Transaction deployment time (t1): The Unix time when the transaction was deployed.
- Transaction completion time (t2): The Unix time when the blockchain confirmed the transaction.
- The performance metrics were calculated as follows:
- Execution Time: For each set of transactions, the execution time is the total time (in seconds) taken by the blockchain platform to execute and confirm all transactions in the dataset (maximum t2–minimum t1).
- Latency: For each transaction, latency is the difference between the completion time and deployment time (t2–t1). For a set of transactions, the average latency is the mean of all transaction latencies in the dataset.
- Throughput: Measured by the number of successful transactions per second, starting from the deployment time of the first transaction. The average throughput is the mean throughput during the execution time.
- Precision: Total number of correct images/Total number of returned images.
- Recall: Total number of correct images/Total number of images that needed to be recalled.

### 3.3. Experimental Results

**Figure 9:**
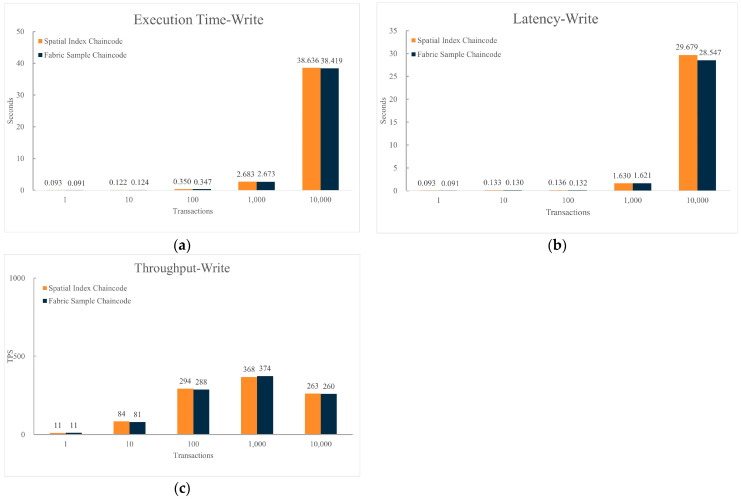
Comparison of chaincode write performance. (**a**) Comparison of execution time for writing data between spatial index chaincode and sample chaincode; (**b**) Comparison of latency for writing data between spatial index chaincode and sample chaincode; (**c**) Comparison of throughput for writing data between spatial index chaincode and sample chaincode.

Figure 9 presents a performance comparison under the same hardware and blockchain network structure, between sending data upload requests to the blockchain network using the Sample chaincode and using the spatial data index chaincode. The number of requests is set to (1, 10, 100, 1000, 10,000).

From Figure 9, it is evident that compared to the Sample chaincode, using the spatial data index chaincode for write operations shows similar performance in terms of execution time, execution delay, and throughput. The execution time and delay of data writing increase with the rising number of transactions in the dataset. When the volume of transactions in the dataset exceeds a certain threshold, the throughput of data writing begins to decline.

**Figure 10:**
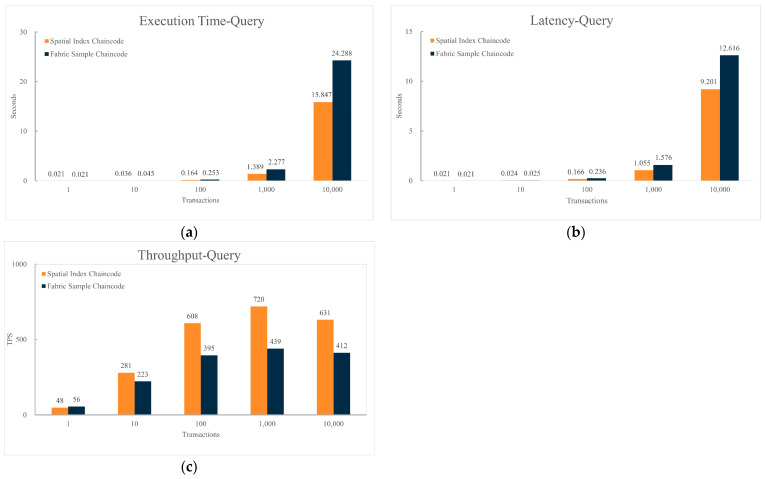
Comparison of chaincode query performance. (**a**) Comparison of execution time for spatial data queries between spatial index chaincode and sample chaincode; (**b**) Comparison of latency for spatial data queries between spatial index chaincode and sample chaincode; (**c**) Comparison of throughput for spatial data queries between spatial index chaincode and sample chaincode.

Figure 10 displays a performance comparison under the same hardware and network structure, between sending spatial data query requests to the blockchain network using the Sample chaincode and using the spatial data index chaincode, with the number of queries set at (1, 10, 100, 1000, 10,000). Due to the lack of support for spatial data queries in the Sample chaincode, to compare the performance of the spatial data index chaincode and the Sample chaincode fairly and effectively, the experiment recorded the query results of the spatial data index chaincode and executed the same number of keyword query requests through the Sample chaincode. This approach allows for a more direct assessment and comparison of the performance differences between the two chaincodes in handling spatial data queries.

From Figure 10, it is apparent that compared to the Hyperledger Fabric Sample chaincode, with increasing data volume, all three performance indicators of the spatial data index chaincode are superior to those of the Sample chaincode. When the number of transactions in the dataset is 1000, compared to the Sample chaincode, the spatial data index chaincode shows a 39% reduction in spatial query execution time and a 33% reduction in execution delay, with a 64% increase in execution throughput. The execution time and delay of queries increase with the number of transactions in the dataset, and the gap between the spatial data index chaincode and the Sample chaincode widens with the increasing number of transactions. The query throughput initially increases with the number of transactions in the dataset, but begins to decline once the number of transactions reaches a certain value.

**Figure 11:**
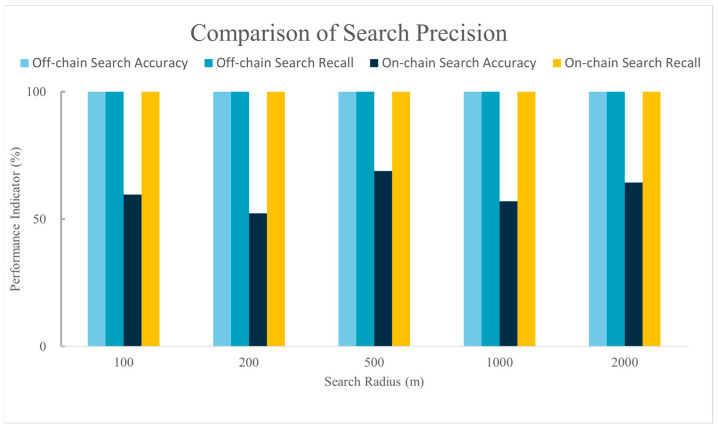
Comparison of spatial data retrieval accuracy of chaincodes.

Figure 11 displays a comparison of retrieval accuracy between the on-chain spatial data query method proposed in this study and the off-chain spatial data query method introduced by Yi Bao [28] under different search radii. Compared to the off-chain query method proposed by Yi Bao and colleagues, the recall rate of our method matches that of the off-chain method, being able to retrieve all data that meet the criteria. However, there is still a gap in retrieval accuracy compared to the off-chain query method.

## 4. Discussion

The spatial data index chaincode, compared to the sample chaincode, showed minimal impact on the blockchain network’s writing performance. This is likely because both types of chaincode do not involve complex computations during data writing; they implement write operations using the blockchain contract function PutState(), resulting in similar performance. However, as the number of transactions in the dataset increases, reaching peaks at 1000 and then declining at 10,000, both writing and querying performance of the consortium chain experience changes. This fluctuation is possibly due to transaction request volumes exceeding the blockchain’s load capacity, leading to network congestion and affecting overall performance [22,29].

The significant difference in read performance can be attributed to several factors:

1. This study designed a Geohash grid-based on-chain spatial data index for remote sensing images. When executing spatial range queries, the algorithm first converts the specified spatial range into the required Geohash grid coverage area. Then, it uses the Geohash codes of the covered grids as keywords to invoke the blockchain contract function GetStateByRange() to query all data within the specified Geohash range. This approach, compared to the sample chaincode which initiates a query for each data item, allows for querying multiple related data entries at once. This significantly reduces the number of requests and network communication overhead, thereby enhancing overall query performance. The comparison of request counts between the Sample chaincode and the spatial index chaincode during spatial data queries is shown in Table 1.

According to the method proposed in this study, remote sensing image data, once encoded, is stored in nodes as Sorted Geohash Tables. A significant advantage of this storage method is that data within the same Geohash grid is stored contiguously on the disk. This continuity means that queries within a specific Geohash grid range can be executed more efficiently in terms of disk I/O operations. It reduces disk seek times and the number of read operations, thereby enhancing the speed of data access.

In terms of retrieval accuracy, to ensure that the blockchain-based spatial index chaincode can retrieve all the remote sensing images that meet the criteria after executing a spatial query, the spatial query method designed in this study prioritizes the recall rate of remote sensing images to prevent missing image data, thereby affecting the credibility of the search results. From the experimental results, our method, like the off-chain query method, can retrieve all the remote sensing images that meet the criteria. However, compared to the off-chain query method, the retrieval accuracy of our method still needs improvement. The off-chain spatial query method connects the blockchain with an external spatial database, using the spatial database’s computational capabilities to query spatial data stored off-chain and compare the query results with the on-chain spatial data metadata. This approach leverages the relatively mature spatial querying technology of spatial databases, thus achieving high recall rates while maintaining high retrieval accuracy and avoiding the direct handling of complex query requests on the blockchain. It somewhat achieves the retrieval of spatial data on the blockchain. However, storing data off-chain still poses serious security and data synchronization issues. Yi Bao had to adopt methods such as periodically scanning data on the blockchain and comparing it with the data in the spatial database, and a two-stage verification mechanism, to ensure the security of off-chain data. In contrast, the on-chain spatial indexing method proposed in this study, although it cannot achieve the accuracy of off-chain retrieval methods, storing spatiotemporal data metadata on the blockchain and directly querying this metadata on the blockchain can effectively leverage the decentralized and tamper-proof characteristics of the blockchain to solve the security and data synchronization issues faced by off-chain spatial queries.

## 5. Conclusions

This paper introduces a blockchain-based method for spatial data retrieval and verification, storing remote sensing images on the secure Hyperledger Fabric consortium chain network. It establishes on-chain spatial indexes for encoded data of remote sensing images within the network, enabling Hyperledger Fabric to support spatial location-based queries. The method uses IPFS within the IPFS-HLF architecture to store original image data, addressing storage limitations of the consortium chain while ensuring data security. The on-chain spatial data index linearizes spatial coordinates using Geohash and integrates with LSM trees for efficient data storage and retrieval. This technique significantly enhances query performance and throughput of the Hyperledger Fabric network, demonstrating superior performance in execution time, latency, and throughput compared to standard keyword searches. Although the blockchain-based spatial data retrieval verification method proposed in this study has shown excellent performance in terms of execution efficiency, latency, and throughput, we still face challenges and limitations in applying this method to real-world application scenarios. First, with the continuous growth of data stored on Hyperledger Fabric, we must address the potential decline in blockchain network performance, a challenge critical to the practical deployment of the method. Moreover, the types of data that need protection in real application scenarios are not limited to image data; vector data, with its complex data structure, poses higher requirements for indexing and querying. This not only increases the complexity of the technical implementation but also challenges the applicability of our existing method.

Considering these factors, our future research will focus on extending the existing method to include indexing and verification of vector data. This means we will explore new data structures and indexing strategies to accommodate the characteristics and needs of vector data. We believe that by continuously optimizing and innovating, we can overcome the limitations of the existing method and expand its applicability in a broader range of spatial data protection applications.

## Figures and Tables

**Table 1:** Comparison of query request numbers.

Query Quantity	Number of Fabric Sample Chaincode Request	Number of Spatial Index Chaincode Request
1	1	1
10	10	4
100	100	42
1000	1000	74
10,000	10,000	127

## Data Availability

The data used to support the findings of this study are available from the corresponding author upon request.
